# Antibacterial Activity and the Mechanism of the Z-Scheme Bi_2_MoO_6_/Bi_5_O_7_I Heterojunction under Visible Light

**DOI:** 10.3390/molecules28196786

**Published:** 2023-09-24

**Authors:** Zhanqiang Ma, Juan Li, Nan Wang, Wei Guo, Kaiyue Zhang

**Affiliations:** 1 College of Agriculture, Henan University of Science and Technology, Luoyang 471000, China; 2 School of Environmental Engineering and Chemistry, Luoyang Institute of Science and Technology, Luoyang 471023, China

**Keywords:** Bi_2_MoO_6_, Bi_5_O_7_I, Z-scheme heterojunction, antibacterial, *Escherichia coli*

## Abstract

Z-scheme Bi_2_MoO_6_/Bi_5_O_7_I heterojunction was constructed by an in situ solvothermal method, which was composed of Bi_2_MoO_6_ nanosheets growing on the surface of Bi_5_O_7_I microrods. The antibacterial activities under illumination towards *Escherichia coli* (*E. coli*) were investigated. The Bi_2_MoO_6_/Bi_5_O_7_I composites exhibited more outstanding antibacterial performance than pure Bi_2_MoO_6_ and Bi_5_O_7_I, and the *E. coli* (10^8^ cfu/mL) was completely inactivated by BM/BI-3 under 90 min irradiation. Additionally, the experiment of adding scavengers revealed that h^+^, •O_2_^−^ and •OH played an important role in the *E. coli* inactivation process. The *E. coli* cell membrane was damaged by the oxidation of h^+^, •O_2_^−^ and •OH, and the intracellular components (K^+^, DNA) subsequently released, which ultimately triggered the apoptosis of the *E. coli* cell. The enhanced antibacterial performance of Bi_2_MoO_6_/Bi_5_O_7_I heterojunction is due to the formation of Z-scheme heterojunction with the effective charge transfer via the well-contacted interface of Bi_2_MoO_6_ and Bi_5_O_7_I. This study provides useful guidance on how to construct Bi_5_O_7_I-based heterojunction for water disinfection with abundant solar energy.

## 1. Introduction

As the world economy continues to develop, water pollution is becoming more and more serious, and the safety of drinking water is becoming increasingly prominent. Water is one of the most abundant resources on earth, and the health risks brought about by the spread of pathogenic microorganisms in drinking water have become a focal point for researchers around the world [1,2]. Many efforts including ultraviolet irradiation, chlorination and ozonation are applied to disinfect most pathogens in drinking water, but the high energy consumption and toxic byproducts restrict their development [3]. It is imperative to develop an environmentally friendly method for the efficient removal of pathogenic microorganisms. Photocatalytic technology, as an emerging advanced oxidation process, has the potential to become a rising star in the field of water disinfection. With the utilized light, the electrons (e^−^) in valence band (VB) of semiconductor are excited to the conduction band (CB) resulting in the production of electron/hole (e^−^/h^+^) pairs. Moreover, the e^−^ can reduce O_2_ to •O_2_^−^ and the h^+^ can oxidate H_2_O or OH^−^ to •OH, respectively. Ultimately, the active species including •O_2_^−^, •OH and h^+^ damage the bacterial cell membranes and contribute to the leakage of intracellular components, accompanied by bacterial inactivation [4,5]. On account of the solar spectrum, the development of stable, efficient and visible-light-responsive photocatalysts is a prerequisite for the application of photocatalytic disinfection techniques.

Bismuth-based semiconductors are considered to be promising photocatalysts by reason of their great variety, favorable stability and low toxicity. Bismuth oxyhalide (BiOX) has a stable structure with an internal electric field (IEF) formed by a (Bi_2_O_2_)^2+^ layer alternately arranged with an I^−^ layer. IEF can be conducive to the migration of photoinduced charges, and BiOX has been widely studied in the fields of pollutant removal from water or air [6,7,8,9], water splitting [10,11], CO_2_ reduction [12,13], nitrogen fixation [14,15], selective oxidation [16,17] and so on. Among them, BiOI is an excellent visible-light-responsive photocatalyst based on the band gap of about 1.8 eV [18,19,20]. On the other hand, the narrow band gap of BiOI results in rapid recombination of photoinduced electrons and holes, in addition to poor redox ability, so the photocatalytic activity is unsatisfactory. Similar to other photocatalytic materials, various strategies such as crystal plane regulation [21,22], element doping [23,24], surface oxygen vacancies [25,26] and construction of heterojunctions [27,28] can be applied to boost the photocatalytic performance of BiOI. Furthermore, the construction of Bi_x_O_y_I_z_ through the bismuth-rich strategy has been shown to be useful for modulating the band structure and enhancing redox capacity [29,30,31]. Furthermore, the bismuth-rich strategy is easy to implement and cannot introduce other elements. Bi_5_O_7_I, as a member of Bi_x_O_y_I_z_, possesses suitable band structure for photocatalytic application, but the limited separation efficiency of photogenerated carriers hinders its performance. Therefore, it is urgent to seek suitable modification methods to enhance the photogenerated carrier separation efficiency of Bi_5_O_7_I.

The formation of heterojunctions is a proven effective way to enhance the separation efficiency of photogenerated carriers. Heterojunctions are usually composed of two semiconductors with suitable band structures, which can effectively encourage the charge transfer and separation. It is acknowledged that charge transfer pathways such as Type I, Type II, and Z-scheme have been researched widely for different heterojunctions [32,33]. For Z-scheme heterojunction, the e^−^ in the CB of one photocatalyst with more positive potential recombine with the h^+^ in the VB of another photocatalyst with more negative potential, which not only promote the separation of the photoinduced e^−^ and h^+^, but also reserve the higher redox capacity. Based on the band structure of Bi_5_O_7_I, another bismuth-based semiconductor material Bi_2_MoO_6_ comes into view. Bi_2_MoO_6_ is an Aurivilius oxide photocatalyst with layer structure consisting of a [Bi_2_O_2_]^2+^ layer and a [MoO_4_]^2−^ layer. Due to its high photooxidation potential, appropriate band structure and environmental friendliness, Bi_2_MoO_6_ shows promise for building a heterojunction with Bi_5_O_7_I, thereby improving photocatalytic performance. So far, the application of the Bi_2_MoO_6_/Bi_5_O_7_I heterojunction as a photocatalytic antibacterial has not been reported.

This work focuses on the synthesis, characterization and photocatalytic antibacterial activity of the Bi_2_MoO_6_/Bi_5_O_7_I heterojunction. The photocatalytic antibacterial activity was evaluated through the inactivation of *Escherichia coli* (*E. coli*) under illumination. The inactivation mechanism for *E. coli* with the Bi_2_MoO_6_/Bi_5_O_7_I heterojunction was also illustrated.

## 2. Results and Discussion

### 2.1. Material Characterization

**Figure 1:**
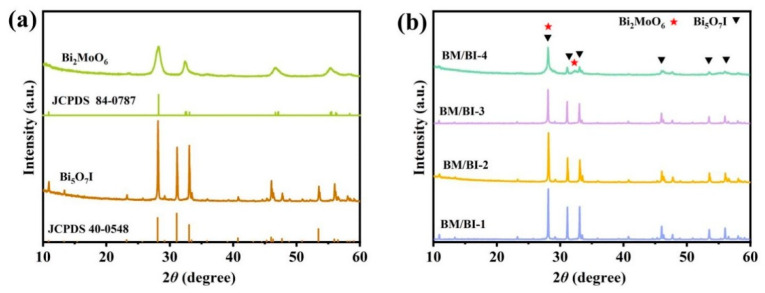
XRD patterns of synthesized samples: (**a**) Bi_5_O_7_I and Bi_2_MoO_6_, (**b**) BM/BI composites.

The XRD patterns of Bi_5_O_7_I, Bi_2_MoO_6_ and BM/BI composites are presented in Figure 1. The diffraction peaks of Bi_5_O_7_I in Figure 1a at 28.1°, 31.1°, 33.1°, 46.0°, 53.5° and 56.0° are in good agreement with the (312), (004), (204), (205), (604), (316), and (912) planes of orthorhombic Bi_5_O_7_I (JCPDS 40-0548) [34]. As for pure Bi_2_MoO_6_, the diffraction peaks can be identified at 28.3°, 32.4°, 46.6° and 55.3° corresponding to the (131), (002), (202) and (331) planes of orthorhombic Bi_2_MoO_6_ (JCPDS 84-0787) [35]. In Figure 1b, the corresponding diffraction peaks of Bi_2_MoO_6_ cannot be recognized for BM/BI-1, BM/BI-2 and BM/BI-3 XRD patterns, but the peak intensities become weaker with the increasing amount of Bi_2_MoO_6_. As for BM/BI-4, the weak peak at 32.4° can be discovered, which corresponds to the (002) plane of Bi_2_MoO_6_, and the peak at 28.1° is broadened, which may be because it is composed of the characteristic peaks of Bi_5_O_7_I (28.1°) and Bi_2_MoO_6_ (28.3°).

**Figure 2:**
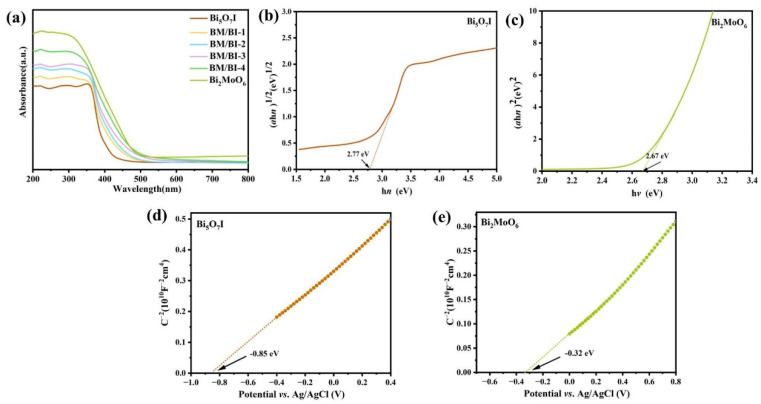
UV–vis diffuse reflectance spectra (**a**) of synthesized samples, Tauc plots (**b**,**c**) and M-S plots (**d**,**e**) of Bi_5_O_7_I and Bi_2_MoO_6_.

The optical absorption property is an important factor affecting the photocatalytic performance of semiconductor materials. The UV–vis diffuse reflectance spectra (UV–vis DRS) of the Bi_5_O_7_I, Bi_2_MoO_6_ and BM/BI composites are exhibited in Figure 2a. Pure Bi_5_O_7_I showed the absorption edge at 450 nm. With the incorporation of Bi_2_MoO_6_, the red shift could be discovered for the absorption edges of the BM/BI composites, which could be conducive to improving the production of photoinduced carriers. As displayed in Figure 2b,c, the band gap energies (*E*_g_) of Bi_5_O_7_I and Bi_2_MoO_6_ are 2.77 and 2.67 eV, respectively. To determine the band structure of Bi_5_O_7_I and Bi_2_MoO_6_, Mott–Schottky (M-S) plots were measured (Figure 2d,e). Bi_5_O_7_I and Bi_2_MoO_6_ are both n-type semiconductors in accordance with the positive slope of M-S plots. As a result, the flat band potentials (*E*_fb_) of Bi_5_O_7_I and Bi_2_MoO_6_ are at −0.85 and −0.32 eV (vs. Ag/AgCl), which can be converted to −0.65 and −0.12 eV (vs. NHE) according to the formula *E*_NHE_ = *E*_Ag/AgCl_ + 0.197 [36]. The *E*_fb_ is usually positive by 0.1 eV over the conduction band potential (*E*_CB_) [37], so the *E*_CB_ of Bi_5_O_7_I and Bi_2_MoO_6_ are estimated to be −0.75 and −0.22 eV (vs. NHE). Consequently, the valence band potentials (*E*_VB_) of Bi_5_O_7_I and Bi_2_MoO_6_ were calculated to be 2.02 and 2.45 eV on account of the formula *E*_CB_ = *E*_VB_ − *E*_g_.

**Figure 3:**
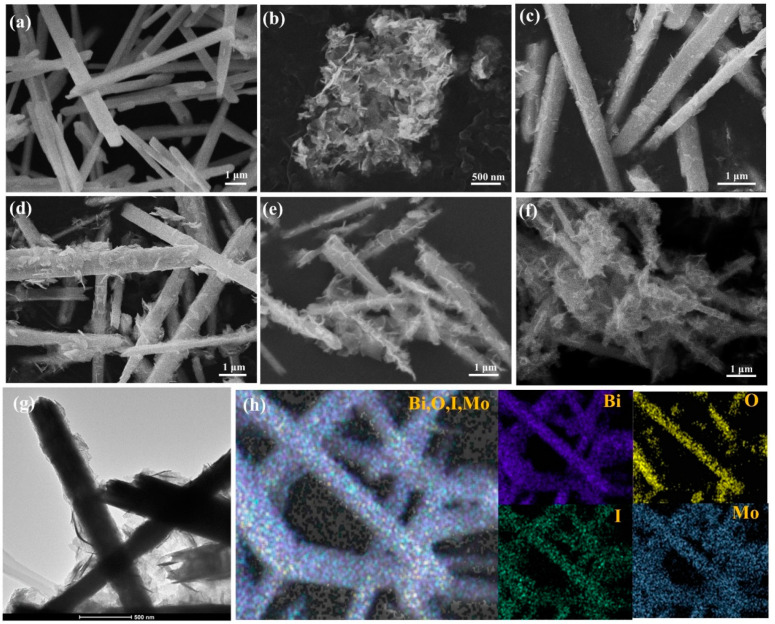
SEM images of Bi_5_O_7_I (**a**), Bi_2_MoO_6_ (**b**), BM/BI-1 (**c**), BM/BI-2 (**d**), BM/BI-3 (**e**) and BM/BI-3 (**f**); TEM image of BM/BI-3 (**g**) and elemental mapping images of BM/BI-3 (**h**).

SEM images of the Bi_5_O_7_I, Bi_2_MoO_6_ and BM/BI composites were measured to determine their micromorphology. Bi_5_O_7_I is composed of uniform microrods with width of 200–800 nm and length of 2–8 µm (Figure 3a). Bi_2_MoO_6_ exhibits the micromorphology of nanosheets with an average size of 300 nm (Figure 3b). For BM/BI composites (Figure 3c–f), Bi_2_MoO_6_ nanosheets growing on the surface of Bi_5_O_7_I microrods can be discovered. As the loading of Bi_2_MoO_6_ increases, the nanosheets covered on the nanorods gradually grow. The TEM image of BM/BI-3 was also displayed in Figure 3g. The nanosheets binding with microrods could be observed in accordance with the results of SEM. In addition, the elemental distribution of BM/BI-3 was obtained by energy disperse spectroscopy (EDS). In Figure 3h, Bi, O, I and Mo elements can be detected and they are evenly distributed, further confirming that Bi_5_O_7_I and Bi_2_MoO_6_ were successfully combined together.

**Figure 4:**
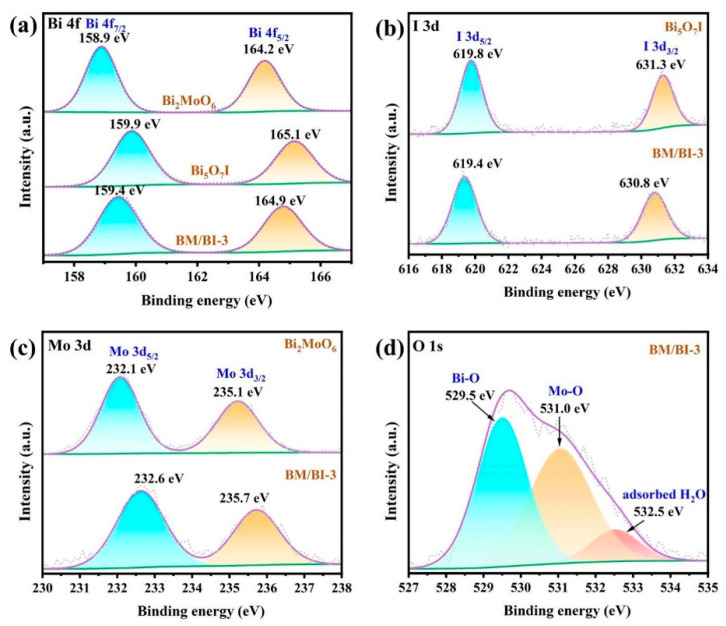
XPS spectra of Bi_5_O_7_I, Bi_2_MoO_6_ and BM/BI-3: Bi 4f (**a**), I 3d (**b**), Mo 3d (**c**), O 1s (**d**).

The element compositions and chemical states of Bi_5_O_7_I, Bi_2_MoO_6_ and BM/BI-3 were analyzed through the XPS measurement. In Figure S1, the survey spectrum confirmed the presence of Bi, I, O and Mo elements, which is in agreement with the elemental mappings of EDS. As exhibited in Figure 4a, the Bi 4f spectrum of BM/BI-3 is composed of two peaks at 159.4 and 164.9 eV, which are correlated with the Bi 4f_7/2_ and Bi 4f_5/2_ of Bi^3+^ [38,39]. In the high resolution XPS spectrum of I 3d for BM/BI-3 (Figure 4b), two peaks at 619.4 and 630.8 eV can be obviously detected, which are assigned to the I 3d_5/2_ and I 3d_3/2_, respectively [34]. Two peaks at 232.6 eV and 235.7 eV can be detected in the Mo 3d spectrum of BM/BI-3 (Figure 4c), corresponding to Mo 3d_5/2_ and Mo 3d_3/2_ of Mo^6+^ from the Bi_2_MoO_6_ [40]. In addition, the O 1 s spectrum of BM/BI-3 (Figure 4d) can be deconvoluted into three peaks at 529.5, 531.0 and 532.5 eV, which are attributed to the Bi-O, Mo-O and the adsorbed H_2_O on the surface, respectively [41]. Compared with pure Bi_2_MoO_6_, the Bi 4f and Mo 3d peaks of BM/BI-3 shifted to a higher energy region, indicating a decrease in the electron density in Bi_2_MoO_6._ Meanwhile, the Bi 4f and I 3d peaks of BM/BI-3 shifted to lower energy region in contrast with pure Bi_5_O_7_I, suggesting an increase in the electron density in the Bi_5_O_7_I. The results demonstrated the formation of the heterojunction and suggested the migration of electrons from Bi_2_MoO_6_ to Bi_5_O_7_I.

**Figure 5:**
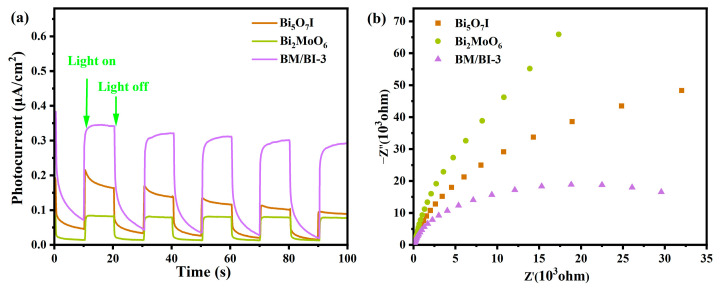
Transient photocurrent response (**a**) and EIS (**b**) of Bi_5_O_7_I, Bi_2_MoO_6_ and BM/BI-3.

To illustrate the separation efficiency of photoinduced carriers, transient photocurrent response and electrochemical impedance spectra (EIS) could be performed. In Figure 5a, the photocurrent can be detected during each light on for Bi_5_O_7_I, Bi_2_MoO_6_ and BM/BI-3, but the photocurrent intensity of BM/BI-3 was higher than that of Bi_5_O_7_I and Bi_2_MoO_6_, indicating the optimal photoinduced carrier separation efficiency of BM/BI-3. Compared with Bi_5_O_7_I and Bi_2_MoO_6_, BM/BI-3 exhibits a lower arc radius in EIS plots (Figure 5b), suggesting more effective separation of photoinduced carriers. Due to the construction of the Bi_5_O_7_I/Bi_2_MoO_6_ heterojunction, the contact interface would facilitate the migration of photoinduced electrons and holes between Bi_5_O_7_I and Bi_2_MoO_6_, and the recombination of photoinduced carriers is successfully suppressed.

### 2.2. Photocatalytic Antibacterial Activity

**Figure 6:**
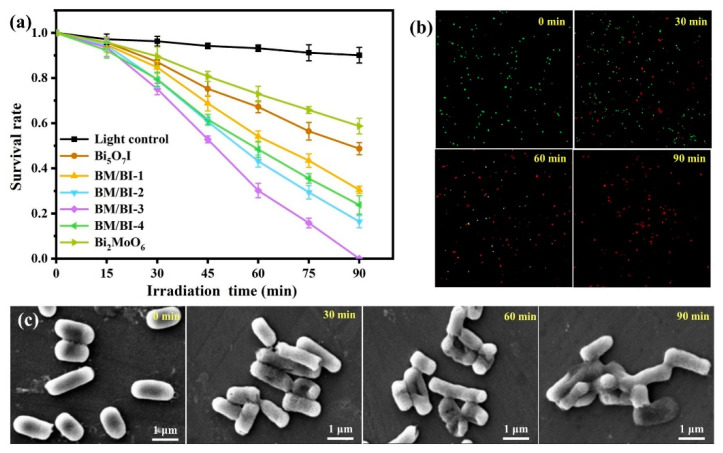
Photocatalytic antibacterial activities toward *E. coli* of synthesized samples (**a**), LSCM images of stained *E. coli* (**b**) and SEM images of *E. coli* cells (**c**) treated by BM/BI-3 under different irradiation time.

To evaluate the photocatalytic performance of fabricated materials, inactivation of *E. coli* under illumination was accomplished and the results were displayed in Figure 6a. Under visible light without synthesized materials, only a slight decrease occurred for the survival rate of *E. coli*, signifying the effect of visible light on *E. coli* is limited. As for Bi_5_O_7_I and Bi_2_MoO_6_ under 90 min illumination, the survival rates of *E. coli* were 48.7% and 58.7%, respectively. The enhanced destructive abilities to *E. coli* can be detected for the Bi_2_MoO_6_/Bi_5_O_7_I composites. Furthermore, BM/BI-3 displayed the optimal photocatalytic performance to inactivate *E. coli* and all *E. coli* were inactivated after 90 min illumination. The antibacterial activities of synthesized samples under dark were also determined and the results were exhibited in Figure S2. Without irradiation, the antibacterial performances of the Bi_5_O_7_I, Bi_2_MoO_6_ and Bi_2_MoO_6_/Bi_5_O_7_I composites were inadequate in 90 min. The synergistic effects of the Bi_2_MoO_6_/Bi_5_O_7_I composite and visible light are favorable for the inactivation of bacteria.

The dead/live *E. coli* cells can be identified by LSCM. Stained by propidium iodide (PI) and SYTO9, live *E. coli* cells glow green fluorescent, while dead display red fluorescent [42,43]. The *E. coli* cells treated by BM/BI-3 under visible light were stained and observed as shown in Figure 6b. With the extension of the illumination time, the number of green dots progressively reduced while red dots increased, indicating that *E. coli* cells were progressively experiencing apoptosis. For the inactivation of *E. coli* by BM/BI-3, the growth of colonies corresponding to the plate count are exhibited in Figure S3 in the supporting information. The number of colonies was gradually reduced as irradiation time increased, which was in agreement with the results of the fluorescence staining measurements. SEM was carried out to investigate the morphological changes in BM/BI-3-treated *E. coli* cells. As exhibited in Figure 6c the untreated *E. coli* cells were bluntly rounded and rod-shaped at both ends and the cell surface was intact. With illumination, wounds appeared on the surface of some cells and gradually became more severe with prolonged irradiation. Furthermore, wounds caused the intracellular components to release and the cell to collapse, and the deactivated cells tended to clump together.

**Figure 7:**
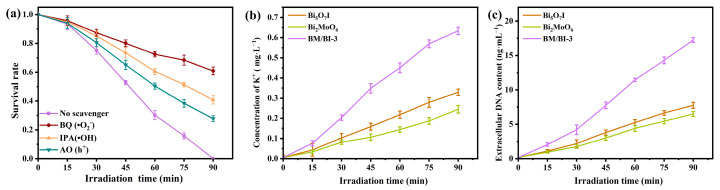
Photocatalytic antibacterial activities of BM/BI-3 with different scavengers (**a**), concentration of leaked K^+^ (**b**), and DNA (**c**) from *E. coli* treated by Bi_5_O_7_I, Bi_2_MoO_6_ and BM/BI-3 under illumination.

Photoinduced electrons (e^−^) and holes (h^+^) can be originated from semiconductor materials under photoexcitation, and subsequently active species including •O_2_^−^ and •OH are also produced. To inactivate the bacteria, h^+^, •O_2_^−^ and •OH can destroy the membrane permeability of *E. coli* cell by means of oxidation, thereby leading to the cell apoptosis [44,45]. In order to analyze the involvement of different active species for *E. coli* inactivation with BM/BI-3, three scavengers including ammonium oxalate (AO, 5 mM), p-benzoquinone (BQ, 5 mM) and isopropanol (IPA, 5 mM) were added for detecting h^+^, •O_2_^−^ and •OH, respectively. In Figure 7a, the antibacterial activity of BM/BI-3 decreased with adding BQ, AO or IPA, indicating that h^+^, •O_2_^−^ and •OH contributed for the *E. coli* inactivation, and the sequence of the effect was •O_2_^−^ > •OH > h^+^.

The active species containing h^+^, •O_2_^−^ and •OH destroy the membrane of the *E. coli* cell, and the wounds on the cell membrane may bring about the release of intracellular components. K^+^ is one of the important intracellular components of *E. coli*, and the extracellular K^+^ concentration of *E. coli* solution was measured at different illumination time (Figure 7b). As treated by Bi_5_O_7_I, Bi_2_MoO_6_ or BM/BI-3, the extracellular K^+^ concentration increased gradually, suggesting that the leakage of K^+^ enhanced as the irradiation time was prolonged. Additionally, the increase of the extracellular K^+^ concentration induced by BM/BI-3 was the most significant, indicating the strong destructive effect of BM/BI-3. The loss of intracellular nucleic acid is lethal for *E. coli* [46], so it is more meaningful to determine the extracellular DNA content of *E. coli* solution (Figure 7c). The extracellular DNA induced by BM/BI-3 was more than that by Bi_5_O_7_I and Bi_2_MoO_6_ under the same illumination time. With the destruction of active species, the wounds on the *E. coli* cell membrane appeared and the intracellular components released, which triggered the *E. coli* cells to ultimately experience apoptosis. Additionally, the destructive ability of active species produced by BM/BI-3 was greater than those produced by Bi_5_O_7_I or Bi_2_MoO_6_.

To further verify whether *E. coli* was completely inactivated after the photocatalytic treatment, the bacterial regrowth experiment was performed. After the photocatalytic antibacterial experiment, BM/BI-3 was removed from the *E. coli* solution. The *E. coli* solution was stored under dark for 4 h and then was directly coated on the LB plate and incubated at 37 °C for 24 h. It was discovered that there was no *E. coli* colony on the plate, demonstrating that photocatalytic disinfection by BM/BI-3 caused irreversible destruction of *E. coli*. The result is consistent with other photocatalytic disinfection reports [47,48].

### 2.3. Mechanism of Improved Photocatalytic Antibacterial Activity for Bi_2_MoO_6_/Bi_5_O_7_I Heterojunction

**Figure 8:**
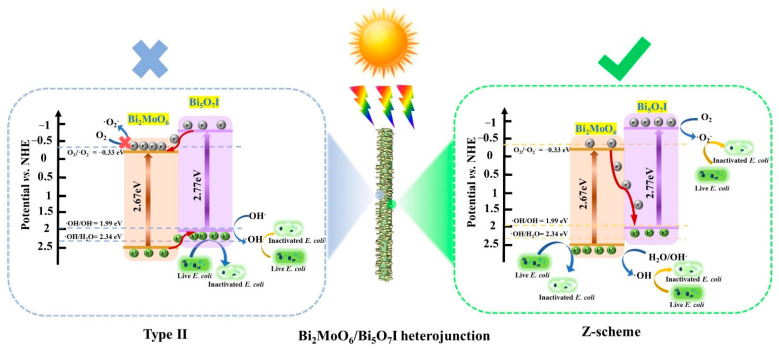
Schematic diagrams for Type II and Z-scheme charge transfer mechanism of the Bi_2_MoO_6_/Bi_5_O_7_I heterojunction.

The charge transfer mechanism of the Bi_2_MoO_6_/Bi_5_O_7_I heterojunction was summarized based on the aforementioned analysis. As exhibited in Figure 8, if Bi_2_MoO_6_ and Bi_5_O_7_I formed the traditional type-II heterojunction, the e^−^ in the CB of Bi_5_O_7_I would transfer to that of Bi_2_MoO_6_ because the *E*_CB_ of Bi_5_O_7_I (−0.75 eV) is more negative than Bi_2_MoO_6_ (−0.22 eV). Simultaneously, the h^+^ would migrate from the VB of Bi_2_MoO_6_ to that of Bi_5_O_7_I based on the more positive *E*_VB_ of Bi_2_MoO_6_ (2.45 eV). But the accumulated e^−^ in the CB of Bi_2_MoO_6_ are unable to convert O_2_ to •O_2_^−^ based on the facts that the *E*_CB_ of Bi_2_MoO_6_ (−0.22 eV) is more positive than the potential of O_2_/•O_2_^−^ (−0.33 eV vs. NHE) [49,50]. According to the results of adding scavengers, •O_2_^−^ participated in the *E. coli* inactivation with BM/BI-3, so it is unreasonable for the type-II charge transfer mechanism. Consequently, a Z-scheme charge transfer mechanism of the Bi_2_MoO_6_/Bi_5_O_7_I heterojunction was put forward in Figure 8. The e^−^ in the CB of Bi_2_MoO_6_ migrated to the VB of Bi_5_O_7_I and recombined with the h^+^, resulting in the accumulation of surplus e^−^ in the CB of Bi_5_O_7_I and h^+^ in the VB of Bi_2_MoO_6_, respectively. The e^−^ in the CB of Bi_5_O_7_I could interact with O_2_ to produce •O_2_^−^, since the *E*_CB_ of Bi_5_O_7_I (−0.75 eV) is more negative than the potential of O_2_/•O_2_^−^ (−0.33 eV vs. NHE). Meanwhile, the h^+^ in the VB of Bi_2_MoO_6_ could interact with H_2_O or OH^−^ to generate •OH, based on the fact that the *E*_VB_ of Bi_2_MoO_6_ (2.45 eV) is more positive than the potentials of •OH/OH^−^ (1.99 eV vs. NHE) [51] and •OH/H_2_O (2.34 eV vs. NHE) [52]. Under the oxidation of h^+^, •O_2_^−^ and •OH, the membrane permeability of the *E. coli* cell was damaged and the release of intracellular components subsequently happened, which ultimately triggered the apoptosis of the *E. coli* cell.

## 3. Experiment Section

### 3.1. Synthesis of Materials

Add 5 mmol Bi(NO_3_)_3_·5H_2_O into 30 mL distilled water and stir magnetically until uniform. Dissolve 5 mmol KI into 30 mL distilled water and stir magnetically until dissolved. Then, add KI solution dropwise to Bi(NO_3_)_3_ solution under continue stirring for 1 h, and the pH of mixed liquor was disposed to 12.5 with dropping NaOH solution (2 mol L^−1^). After stirring for 30 min, the suspension was poured into a hydrothermal autoclave and heated at 160 °C for 10 h. When the autoclave cooled to room temperature, centrifugal washing with distilled water and ethanol was carried out and the precipitate was dried and collected as Bi_5_O_7_I.

**Figure 9:**
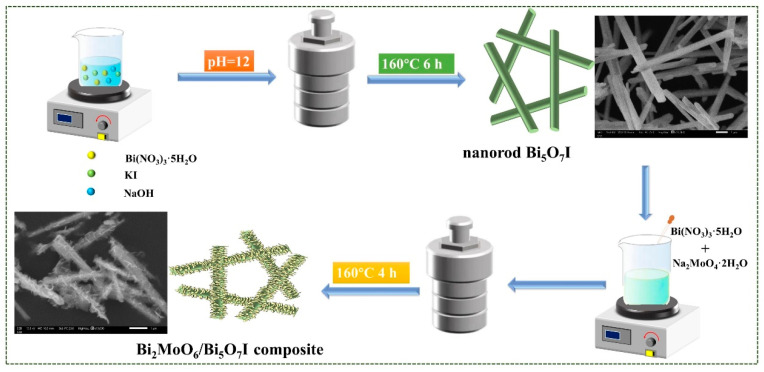
Schematic diagram for construction of the Bi_2_MoO_6_/Bi_5_O_7_I composite.

The suspension composed of 500 mg Bi_5_O_7_I and 50 mL deionized water was sonicated for 1 h. A certain amount of NaMoO_4_·2H_2_O and Bi(NO_3_)_3_·5H_2_O (molar ratio 1:2) were dissolved into·20 mL ethylene glycol. The above solution was dropwise added into Bi_5_O_7_I dispersion under ultrasonication for 30 min and stirred for another 30 min, which was poured into a hydrothermal autoclave with heat at 160 °C for 4 h. After cooling, centrifugal washing and drying were followed up and the collected powder was the Bi_2_MoO_6_/Bi_5_O_7_I composite. According to the above process, the amount of Bi(NO_3_)_3_·5H_2_O added was 0.01 and 0.02, 0.03, 0.04 mmol, and the products were named BM/BI-1, BM/BI-2, BM/BI-3 and BM/BI-4, respectively. The schematic diagram for synthesis of Bi_2_MoO_6_/Bi_5_O_7_I composite is shown in Figure 9.

### 3.2. Characterization and Photoelectrochemical Measurement

Powder X-ray diffraction (XRD) was taken on a Bruker D8A X-ray powder diffractometer with Cu Kα radiation at 2θ = 10~60°. UV–vis diffuse reflectance spectra (DRS) were operated on a Shimadzu UV-2600i spectrophotometer with BaSO_4_ as a reference. The micromorphology was obtained using a scanning electron microscope (SEM, JSM-IT200, Japan Electronics Co., Ltd., Tokyo, Japan) and transmission electron microscope (TEM, JEM-2100, Japan Electronics Co., Ltd., Tokyo, Japan). The elemental mapping images were achieved by energy dispersive spectrometer (EDS, JED-2300, Japan Electronics Co., Ltd., Tokyo, Japan) coupled with SEM. X-ray photoelectron spectroscopy (XPS) was measured by Kratos AXIS NOVA spectrometer (Kratos Analytical, Ltd., Manchester, UK).

The electrochemical experiments were performed on an electrochemical workstation (CHI660E, Shanghai Chenhua Instrument Co., Ltd., Shanghai, China) using the three-electrode system. The synthesized powder (10 mg) was mixed with 1 mL ethanol and 20 μL Nafion solution (5%) under ultrasonic and then coated on the FTO glass as the working electrode. The Ag/AgCl electrode was selected as the reference electrode and the Pt wire was chosen as the counter electrode. The Na_2_SO_4_ solution (0.1 M) was taken as the electrolyte for the photoelectrochemical experiments. Additionally, transient photocurrent response tests were performed under 300W Xe lamp irradiation with 420 nm cut-off filter. Electrochemical impedance spectroscopy (EIS) and Mott–Schottky (M-S) plots were measured in the dark. Moreover, EIS were investigated in a frequency range from 1 Hz to 10 kHz and M-S plots were studied at the frequency of 1000 Hz.

### 3.3. Photocatalytic Inactivation of E. coli

The antibacterial performance with visible light was investigated by the inactivation of *E. coli* (ATCC 8739, Shanghai Beinuo Biotechnology Co., Ltd. Shanghai, China). The operational vessel must be autoclaved at 121 °C for half an hour and the following antimicrobial procedures need to be fulfilled in the sterile environment. A 300 W Xe lamp with a 420 nm cut-off filter was adopted as visible light. The antibacterial photocatalytic experiment was performed by 50 mL *E. coli* solution (10^8^ cfu/mL) with 20 mg photocatalyst. At 15 min intervals, 3 mL suspension was taken out and centrifuged. The supernatants were employed to determine intracellular components (K^+^ and DNA) release. The precipitate was rinsed with a PBS buffer solution three times and suspended in a PBS buffer solution. To determine the cell density of *E. coli*, the plate count method was applied. An amount of 1 mL of the above solution was diluted with a gradient of 10^−1^ and coated on a Luria–Bertani (LB) plate with incubation at 37 °C for 24 h. The number of colonies on the plate was counted to evaluate the antibacterial performance of the synthesized samples under different irradiation times.

### 3.4. Fluorescence Microscopy Assays and Microstructure of E. coli

To further identify the survival state, laser scanning confocal microscopy (LSCM) and scanning electron microscope (SEM) were operated. To identify the dead/live *E. coli* cells, the bacteria were stained by propidium iodide (PI) and SYTO9. The PI solution (5 μg/mL) and SYTO9 solution (5 μg/mL) were mixed with a volume ratio of 1:1. The PBS buffer solution with *E. coli* and the PI/SYTO9 solution were uniformly mixed and reacted in the dark for 10 min. The stained *E. coli* was then centrifuged and washed with PBS three times and observed by LSCM.

To study the morphological change in *E. coli* cells during the photocatalytic antibacterial process, 2.5% (*v*/*v*) glutaraldehyde solution was used to fix the *E. coli* cells at 4 °C for 6 h. Next, after washing with a PBS buffer solution, the *E. coli* cells were gradually dehydrated with ethanol solution (30%, 50%, 70%, 90% and 100%) for 10 min each time and tert-butanol for 20 min. Eventually, SEM was operated to observe the microstructure of the *E. coli* cells.

### 3.5. Measurement of Intracellular Components Leakage

The leakage of intracellular components was determined using the supernatant from the *E. coli* suspension at different irradiation times. The released K^+^ from *E. coli* cells was detected by inductively coupled plasma optical emission spectroscopy (ICP-OES). Extracellular DNA content was determined by NanoDrop One at 260 nm.

## 4. Conclusions

In summary, a Bi_2_MoO_6_/Bi_5_O_7_I heterojunction was constructed via an in situ solvothermal process for antibacterial application. The Z-scheme charge transfer through the interface of Bi_2_MoO_6_ and Bi_5_O_7_I constrained the recombination of photoinduced carriers and enhanced their antibacterial performance under visible light. On the basis of the experiment of adding scavengers, h^+^, •O_2_^−^ and •OH played an important role in *E. coli* inactivation. The membrane permeability of the *E. coli* cell was damaged by the oxidation of h^+^, •O_2_^−^ and •OH, and the intracellular components (K^+^, DNA) subsequently released, which ultimately triggered the apoptosis of the *E. coli* cell. This study offers an opportunity to construct a Z-scheme Bi_5_O_7_I-based heterojunction for water disinfection with abundant solar energy.

## Data Availability

The data of the study can be provided by corresponding author upon reasonable request.

## Supplementary Materials

The following supporting information can be downloaded at: https://www.mdpi.com/article/10.3390/molecules28196786/s1, Figure S1: XPS survey spectrum of BM/BI-3; Figure S2: Antibacterial activities of synthesized samples under dark; Figure S3: Bacterial colonies of re-cultured *E. coli* treated with BM/BI-3 under different irradiation time.

## Sample Availability

Samples of the compounds are available from the authors.
