# Supplementary material for: Antibacterial Activity and the Mechanism of the Z-Scheme Bi2MoO6/Bi5O7I Heterojunction under Visible Light
Source: Molecules. 2023 Sep 24;28(19):6786. doi: 10.3390/molecules28196786 (PMC10574076; doi:10.3390/molecules28196786)
Supplement: Supplementary file 1 [file molecules-28-06786-s001.zip › molecules-2608981-supplementary.pdf]

Supporting Information

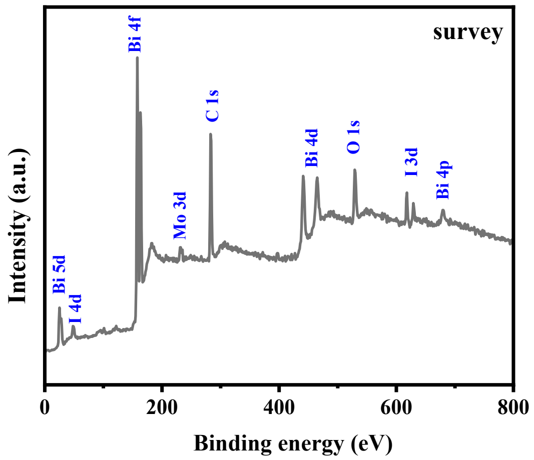

Fig. S1 XPS survey spectrum of BM/BI-3.

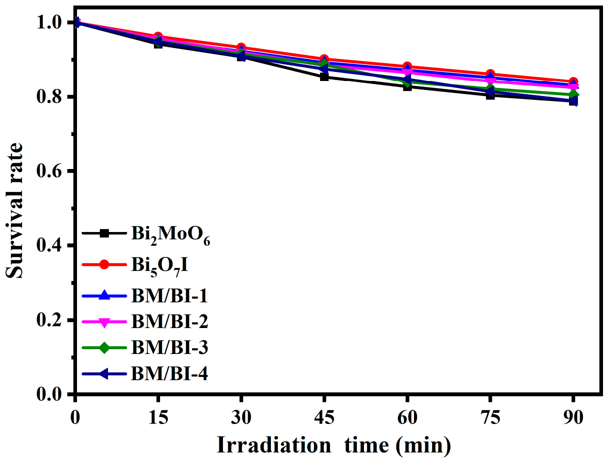

Fig. S2 Antibacterial activities of synthesized samples under dark.

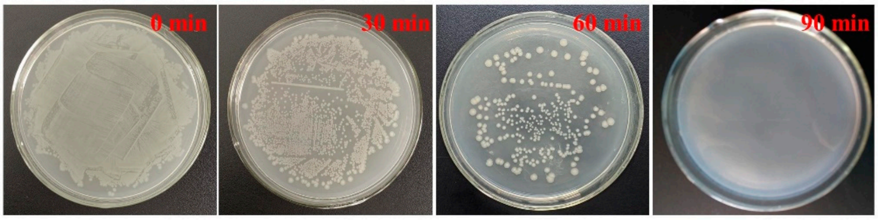

Fig. S3. Bacterial colonies of re-cultured *E. coli* treated with BM/BI-3 under different irradiation time.
